# Teaching Musculoskeletal Physical Diagnosis Using A Web-based Tutorial and Pathophysiology-Focused Cases

**DOI:** 10.3885/meo.2009.Res00301

**Published:** 2009-09-28

**Authors:** Renee F Modica, Josef G Thundiyil, Calvin Chou, Mohammad Diab, Emily Von Scheven

**Affiliations:** * Department of Pediatrics, University of Florida, Gainesville, Florida, USA; † Department of Emergency Medicine, Orlando Healthcare Systems, Orlando, Florida, USA; ‡ Department of Internal Medicine, University of California-San Francisco, San Francisco, California, USA; § Department of Orthopedics, University of California-San Francisco, San Francisco, California, USA; ǁ Department of Pediatrics, University of California-San Francisco, San Francisco, California, USA

**Keywords:** cases, curriculum, musculoskeletal, OSCE, physical exam, tutorial, website

## Abstract

**Objective:**

To assess the effectiveness of an experimental curriculum on teaching first-year medical students the musculoskeletal exam as compared to a traditional curriculum.

**Background:**

Musculoskeletal complaints are common in the primary care setting. Practitioners are often deficient in examination skills and knowledge regarding musculoskeletal diseases. There is a lack of uniformity regarding how to teach the musculoskeletal examination among sub-specialists. We propose a novel web-based approach to teaching the musculoskeletal exam that is enhanced by peer practice with pathophysiology-focused cases. We sought to assess the effectiveness of an innovative musculoskeletal curriculum on the knowledge and skills of first-year medical students related to musculoskeletal physical diagnosis as compared to a traditional curriculum. The secondary purpose of this study was to assess satisfaction of students and preceptors exposed to this teaching method.

**Methods:**

This quasi-experimental study was conducted at a single LCME-accredited medical school and included a convenience sample from 2 consecutive classes of medical students during the musculoskeletal portion of their physical diagnosis class. We conducted a needs assessment of the traditional curriculum used to teach musculoskeletal examination. The needs assessment informed the development of an experimental curriculum. One class (control group) received the traditional curriculum while the second class (experimental group) received the experimental curriculum, consisting of a web-based musculoskeletal tutorial, pathophysiology-focused cases, and facilitator preparation. We used multiple-choice questions and musculoskeletal OSCE scores to assess differences between knowledge and skills in the 2 groups.

**Results:**

The sample consisted of 140 students in each medical school class. There were no statistically significant differences between the 2 groups. One hundred seven students from the control group and 120 students from the experimental group took the multiple-choice examination. The average score was 66% (95% CI= 59.7–72.3) for the control group and 66% (95% CI = 60.5–71.5) for the experimental group. There was no difference between the median musculoskeletal OSCE scores between the 2 groups. The experimental group was satisfied with the new teaching method and gained the additional benefit of a persistent resource.

**Conclusions:**

This web-based experimental curriculum was as effective as the traditional curriculum for teaching the musculoskeletal exam. Additionally, users were satisfied with the web-based training and benefited from a persistent resource.

**Acronyms**
MCE: Multiple Choice Examination
M-OSCE: Musculoskeletal-Objective Structured Clinical Examination
PFCs: Pathophysiology-Focused Cases
SQs: Satisfaction Questionnaires
UCSF: University of California, San Francisco
VAS: Visual Analog Scale
WMT: Web-based Musculoskeletal Tutorial

Musculoskeletal complaints are common in the primary care setting.^1^ Arthritis is one of the most prevalent diseases in the United States affecting 43 million Americans (about 1 in 6 people) and 285,000 children.^1^ This rising prevalence is a growing societal burden.^1^–^3^ The associated economic and social consequences make improved teaching of the musculoskeletal exam an important challenge for medical educators.^1^,^4^ Despite this growing prevalence, musculoskeletal complaints are under-recognized and poorly addressed by primary care providers, often leading to delayed diagnosis. There is a decreased frequency of documentation by community physicians regarding musculoskeletal complaints compared to other systems.^5^ Residents are often deficient in performing the musculoskeletal examination.^6^,^7^ General Practitioners have identified the need to update their skills in musculoskeletal medicine.^8^

There is both a lack of uniformity and of information among sub-specialists about how to teach the musculoskeletal examination.^3^ A combination of teaching strategies that introduce material in a didactic manner and provide students with self-directed assignments are generally considered superior to using only a single teaching method.^9^ Computer-assisted learning has the potential to provide a standardized, interactive, convenient learning experience to a large number of students over a wide geographical area.^10^ Knowledge is remembered and recalled more effectively with case-based learning.^9^ It is imperative to improve the teaching of the musculoskeletal exam and expose learners to the musculoskeletal examination earlier in their careers.^11^

We sought to assess the effectiveness of an innovative musculoskeletal curriculum compared to that of a traditional curriculum on first-year medical students’ knowledge and skills related to musculoskeletal physical diagnosis. The secondary purpose of this study was to assess students’ and preceptors’ satisfaction when exposed to our experimental teaching method.

## Methods

**Needs Assessment** - We conducted a local needs assessment during the fall semester for the class of 2007 through observation of the traditional curriculum, conducting focus groups with faculty and students, reviewing course evaluations, and examining student performance on prior course examinations and scores on the Musculoskeletal-Objective Structured Clinical Examination (M-OSCE). This information was used to inform the development of an innovative curriculum to address the identified weaknesses. Identified weaknesses included missing subtleties of the exam, uncertainty in performing special tests to elicit pathology and recognizing abnormal physical findings, lack of uniformity and lack of resources. These weaknesses occurred due to poor visualization, instructor variability, and inexpert facilitators. The exam was demonstrated in front of the class in a lecture auditorium. Each year the musculoskeletal exam was taught by a different specialist (e.g., orthopedics, rheumatology, family practice, or sports medicine) who emphasized his or her own sub-specialty. Subsequently, students attended unstructured small group peer practice sessions with inexpert facilitators. Consequently, different aspects of the exam were emphasized depending on the facilitator. Additionally, there was a lack of effective resources available to review the many complex components of the examination in preparation for the M-OSCE.

**Setting** - This study was conducted at a single LCME accredited medical school, the University of California-San Francisco (UCSF), in the United States.

**Study Population** - A convenience sample, determined by the number of students present on day of the study, of all first-year medical students from the classes of 2007 and 2008 during their musculoskeletal physical diagnosis curriculum was used.

**Control Group** - The class of 2007 received the traditional musculoskeletal curriculum during the physical diagnosis course during their first year of medical school. This course was a 16-month longitudinal curriculum conducted once weekly for 2–3 hours. The traditional curriculum consisted of a 1-hour didactic PowerPoint lecture given by an orthopedic sub-specialist. Emphasis was placed on orthopedic maneuvers to detect sports injuries. The musculoskeletal exam was demonstrated to the entire class by the orthopedist on a student volunteer. Subsequently, students participated in small group peer-practice session led by a facilitator, with 8–10 students per facilitator. The facilitators received no training prior to their roles as facilitators. No standardized checklists, references or cases were provided.

**Experimental Group** - The class of 2008 received the experimental musculoskeletal curriculum during their first year of medical school. It consisted of 2 presentation elements, a web-based musculoskeletal tutorial and pathophysiology-focused cases. This experimental curriculum also involved a faculty preparation element.

**Figure 1.**
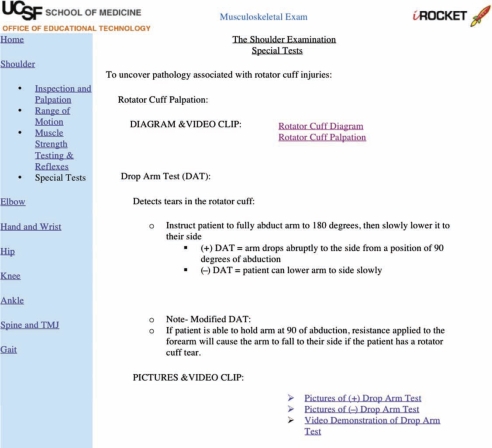
This partial web page shown is a portion of the shoulder exam, special test section of the WMT. Note the navigation toolbar on the left that divides the locomotor system into regions and the region selected has links to the “4-step approach.” A brief definition an description of the exam maneuver is provided. Users could access various links to diagrams, pictures and video clips.

**Figure 2.**
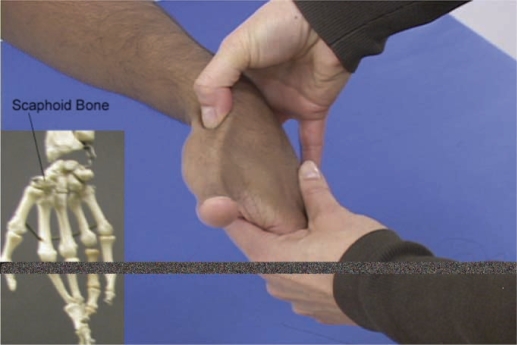
Palpation of body surface anatomy is shown in close up with use of split screen to correlate with skeletal body anatomy. Use of graphics and labels to help orient viewer.

1. Web-based Musculoskeletal Tutorial (WMT)**-**The WMT (created by RFM) used a standard 4-step methodical approach to the musculoskeletal examination applied to each “region of the locomotor system.” The 4-step approach consisted of inspection and palpation, muscle strength testing, range of motion testing, and special tests to elicit pathology. The web page provided a navigation toolbar by region and a general outline for each step, including learning objectives (located on the main introductory page for each region), links to anatomy diagrams, step-by-step text describing some exam maneuvers, and links to pictures, diagrams and videos demonstrating that particular part of the exam (Figure 1). The unique features of the pictures and videos included demonstration of the musculoskeletal examination on a skeleton, on normal patients, and on patients with abnormal physical findings. The bony palpation of surface anatomy was demonstrated in a side-by-side “split-screen” fashion in order to assist correlation of surface anatomy with the underlying bony anatomy (Figure 2).

Additionally, examples of “abnormal physical findings” were included to differentiate from the normal physical findings (not shown). Graphics and labels were applied throughout the video to orient the exam and enhance understanding of the maneuvers. Emphasis was placed on special tests to elicit pathology and abnormal physical exam findings because these topics were identified as areas of particular uncertainty in our needs assessment. Experts in musculoskeletal medicine (including pediatric rheumatology, pediatric orthopedics and family medicine) were consulted for feedback on the content and exam maneuvers. Students were strongly encouraged to preview the website prior to their peer practice sessions. A composite video of the entire exam from the website was shown in place of the traditional didactic lecture so that all students could view the exam on a larger screen and have the opportunity to ask questions. A syllabus was provided that correlated with the components of the video.

2. Pathophysiology Focused Cases (PFCs)-The PFCs were brief clinical case vignettes concentrating on the use of special tests to elicit pathology. These PFCs were used to help structure the peer practice sessions. Each small group reviewed 3 cases: shoulder (impingement), knee (anterior cruciate ligament tear), and back (sciatica). The cases included directed discussion points designed to help review pathology, abnormal findings and differential diagnoses. A checklist, organized according to the 4-step approach with descriptions of the exam maneuvers, was provided for each case during peer practice. The students used the checklist as a guide to perform each exam maneuver. Approximately 40 minutes were allotted for each case.

3. Preparation of facilitators**-**Facilitators were asked to preview the website prior to the peer practice sessions. Additionally, excerpts from the website were shown on the day of the session to review the pertinent physical exam findings of the PFCs with a musculoskeletal exam expert (RFM). All 12 facilitators attended this session. During this session, instructors were able to ask questions and practice their exam maneuvers. Facilitators were provided syllabi to assist their facilitating the discussion points and exam findings of the PFCs. Facilitator standardization was not, however, measured directly.

**Figure 3.**
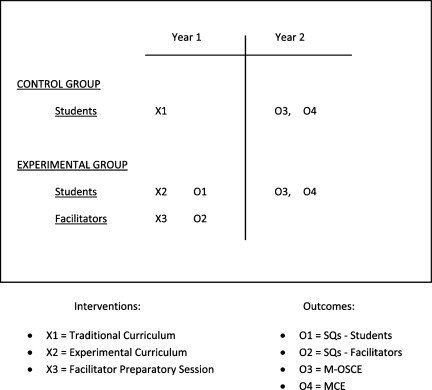
Schematic of Study Design (Quasi-Experimental).

**Research Design** - To assess the efficacy of the experimental curriculum, an intact groups quasi-experimental design was used to compare knowledge, skills and satisfaction of the 2 groups. (Figure 3)

**Outcome Measures** - The primary outcome measures, musculoskeletal knowledge and skills, were assessed using Multiple Choice Examination (MCE) and M-OSCE scores, respectively. The MCE and M-OSCE were implemented and data were collected during the second year of medical school for both groups. The M-OSCE consisted of a shoulder impingement case. Trained standardized patients graded the M-OSCE for the experimental group, whereas an untrained third-party observer was used to grade the control group. A score sheet was used to grade the encounter and physical exam. This score sheet included a more precise set of grading instructions only for the standardized patients who graded the experimental group. Specifically the “checkbox” system (which was used for the control group) was changed to “bubbles” in order to deter graders from checking an intermediate answer. Also, a diagram was added to the score sheet demonstrating how to fill in the bubble correctly vs. incorrectly. Good inter-rater reliability for grading with the bubble system was demonstrated among the standardized patients, whereas inter-rater reliability was not assessed for the untrained third-party observers who graded the control group with the checkbox system. The OSCE exam has been used effectively as a means of assessing clinical skills, particularly in graduate education.^12^ Subsequently, each medical student was given 15 minutes to complete a MCE consisting of 3 physical exam questions. Two different versions (A and B) of the MCE were administered in order to discourage students from discussing their answers to the MCE. Each version consisted of 1 shoulder, 1 hip/back and 1 knee question for a total of 6 different case based questions. Half of each class received version A and the other half received version B. Two faculty members reviewed the MCEs for face validity. The subjects knew that the M-OSCE score was part of the summative grade for their physical diagnosis course, whereas the MCE was only used for research purposes.

The secondary outcome measure, satisfaction, was assessed through the use of anonymous student and faculty Satisfaction Questionnaires (SQs). The SQs were administered immediately after the peer practice sessions to both the teaching faculty and the experimental group and were returned via campus mail. The SQs were not administered to the control group. The SQs were geared to assess subjects’ satisfaction with the individual components of the experimental teaching methods: video portion, WMT, and PFC. The SQs consisted of a 5-point Likert scale, a visual analog scale (VAS), multiple choice questions and opportunity for qualitative comments. Separate versions were administered to faculty and students.

**Statistical Analysis** - Data were analyzed with descriptive methods and the unpaired t-test to compare MCE scores of the 2 groups. A p value of 0.05 was considered statistically significant. We used the groups’ mean M-OSCE scores to compare skills acquisition. Descriptive statistics were used to evaluate attitudes toward the new curriculum in the experimental group. All statistical analyses were conducted using STATA 8.0 and Microsoft Excel 2008. Given a sample size of 100 students in each group, we estimated a power of 80% to detect an effect size of 0.4.

**Institutional Review Board** - IRB approval was obtained prior to implementation.

## Results

There were 141 subjects in each group. The 2 classes were demonstrated to be equivalent based on their year-1 composite including: GPA, MCAT scores, and most demographic characteristics. (Table 1)

**Table 1.** Baseline comparison of study populations

**Demographic Factors**	**Control Group N (%)**	**Experimental Group N (%)**
**N**	141	141
**GENDER**		
Male	58 (41.13)	70 (49.65)
Female	83 (58.87)	71 (50.35)
**AGE (mean)**	24	24
**STATE of RESIDENCE**		
In state	115 (81.56)	114 (80.85)
Out of state	26 (18.44)	27 (19.15)
**ETHNICITY**		
White	66 (46.81)	60 (42.55)
Asian^*^	35 (24.82)	50 (35.46)
Black	9 (6.38)	9 (6.38)
Mexican	8 (5.67)	12 (8.15)
Native	3 (2.13)	0 (0)
Uncertain^*^	20 (14.18)	10 (7.10)
**GPA**		
Overall	3.76	3.77
Science	3.76	3.77
**MCAT**		
Biology	12	11

^*^represents statistically significant differences (p < 0.05)

**Needs Assessment** - The students reported uncertainty in their ability to perform special tests to elicit pathology and ability to recognize an abnormal physical exam finding.

**Knowledge** - One hundred seven students (76%) from the control group and 120 students (85%) from the experimental group completed the MCEs. The control group's average mean score was 60% (95% CI 54.5–65.5), compared to 51% (95% CI 46.5–55.5) for the experimental group. The groups were next compared separately on each of the 3 questions. There were no significant group differences on test scores for the shoulder or knee questions from either version A or version B. There was, however, a group mean difference for the hip/back question on test version A: the mean question score was 55% for the control group (SD = 0.51) and 15% (SD = 0.36) for the experimental group (p = 0.0001). When the hip /back question was eliminated from both versions of the MCE, the control group's average score was 66% (95% CI= 59.7–72.3) and 66% (95% CI = 60.5–71.5) for the experimental group (Table 2).

**Table 2.** Comparison of knowledge and skills outcomes between both groups

**Category**	**Control Group Score (N = 141)**	**95%CI**	**Experimental Group (N = 141)**	**95%CI**
**MCE- N (%)**	107 (76%)		120 (85%)	
Overall test score	60%	54.5–65.5	51%	46.5–55.5
Test score without back question	66%	59.7–72.3	66%	60.5–71.5
**M-OSCE- N (%)**	102 (72%)		124 (85%)	
**Score**	**Percentage of subjects achieving specified score**			
2.5	1%		5.6%	
3	40.2%		59.7%	
3.5	12.7%		6.5%	
4	46.1%		28.2%	

**Skills** - One hundred two students (72%) from the control group and 124 students (85%) from the experimental group completed the M-OSCEs. The median score of the potential 0–5.00 score for both groups was 3. The experimental group performed better on questions related to ‘special tests to elicit pathology’ for the shoulder impingement and drop-arm tests (Table 2).

**Attitudes** - One hundred nine students from the experimental group (77%) and 6 faculty members (60%) who taught the experimental curriculum completed the SQs. The median scores on a 5-point Likert scale of satisfaction (1 = strongly disagree, 5 = strongly agree) are depicted in Table 3. When asked to rank elements in order of usefulness, 67% of students thought that the PFCs were most useful, followed by 19% for the video portion and 14% for the website content. For faculty, 60% thought their preparatory session was most useful, followed by 20% for the website and 20% for the video. The visual analog scale(VAS) means, with potential values from 0 to10, reflecting students’ overall satisfaction with the methods used to teach the musculoskeletal curriculum and confidence of musculoskeletal diseases, were 7.0 and 5.4, respectively. The VAS means for faculty regarding overall satisfaction with the methods used to teach the musculoskeletal curriculum and preparation to teach this curriculum were 6.1 and 5.3, respectively.

**Table 3.** Results for attitude and satisfaction questionnaires in experimental group

**Category**	**Mean Score (SD)**	**N**
	**Students’ Overall Ratings**	
Video	3.84 (0.61)	108
Web	3.85 (0.69)	71
PFC	4.24 (0.59)	104
	**Faculty Overall Ratings**	
Video	3.52 (0.34)	6
Web	3.36 (0.91)	2
PFC	3.55 (0.44)	6
Preparation	3.96 (0.43)	6

Qualitative comments were collected form the experimental group and faculty who taught the experimental curriculum regarding perceived strengths and weaknesses of each curriculum component. Most comments came from a large proportion of the respondents. Students reported that the strengths of the video were its clear comprehensive review, instructive pictures and video, use of the skeleton and normal vs. abnormal findings. Weaknesses included too much information and too rapid a pace. Strengths of the website included the ability to view at home and self-pace, ease in navigation and organization, comprehensive and thorough content, good visual teaching tools, and good resource for future reference. Weaknesses included the length of time needed to complete, difficulty with access, time to upload videos, and information included that will not be tested. Strengths of the PFCs included the relevance and practicality, reinforcement of concepts, correlation with anatomy and pathophysiology, active thinking engaged, group learning, and focus on the special tests to elicit pathology. Weaknesses included the limited knowledge to answer questions, lack of time to complete cases and exam, practicing on normal subjects and no temporal correlation with their gross anatomy coursework. Faculty reported strengths of the experimental curriculum were the availability, clarity, group discussion, realistic cases, and good visuals. Weaknesses included insufficient time to prepare, too long, a preference to teach more basic skills, and too much additional information.

## Conclusions

This is the first interventional study, to our knowledge, to assess the efficacy of a web-based tutorial for teaching musculoskeletal physical diagnosis. Our needs assessment revealed that the traditional curriculum had several important weaknesses. Student evaluations reported a lack of clear goals and objectives, inconsistent content depending on the instructor, poor visualization of exam demonstration and lack of useful resources to review the examination. Focus group data revealed that facilitators felt unprepared to teach the examination and that peer practice sessions were unstructured. Students reported poor understanding of exam maneuvers. M-OSCE performance in the past had been sub-par. There was, therefore, a clear need for improvement in this area.

These weaknesses are identified in many traditionally taught musculoskeletal examination curricula.^13^–^16^ Prior research on this topic has suggested that the use of structured clinical instruction modules can result in reduced demands on medically trained personnel, increased relevance, a multidisciplinary approach, standardization of content, usage of adult learning principles, and the ability to provide direct feedback to students about their clinical skills.^17^ Our educational innovation had the same advantages with the addition of a persistent comprehensive resource, the web based module and video.^18^,^19^

From a knowledge standpoint, the experimental group performed equally as well as the control group. As for skills, tests that were closely tied to the curricular intervention and technically difficult to both teach and perform correctly demonstrated improvement (i.e., special tests for the shoulder exam). Unfortunately, no significant improvements on the M-OSCE measures were obtained. However, limitations to the study must be considered when interpreting these data.

Despite the lack of significant improvement in knowledge and skills outcome measures, both students and faculty expressed many positive comments in the form of qualitative and anecdotal feedback. Specifically, formalized curriculum, use of a standardized approach to the clinical exam, improved visualization of the physical exam through the use of split-screen, close up pictures/video and graphics were all cited as beneficial. Significantly, most negative comments centered on time constraints that limited students’ ability to view the entire video and web module. This was largely due to the meticulous detail present in these teaching tools. Since, the WMT is a tangible educational program for students and faculty to review the musculoskeletal exam, its use as a resource throughout medical training is possibly an additional benefit. PFCs provided structure, relevance, practicality, group learning experience and explanations of exam maneuvers to the peer practice session with improved preparation of the facilitators. VAS data regarding overall satisfaction with the experimental curriculum were positive.

Potential limitations to be considered when interpreting the findings are related to the groups’ nonequivalence and the quasi-experimental study design. Although the individual year-1 composites for both classes were similar, lack of randomization, history threat, or other unmeasured differences may have confounded the results. The use of convenience sampling with compliance rates ranging from 72–85% may have affected the results. However, we have no reason to suspect that subjects who did not complete a portion of the study would have scored differently from one year to the next, causing differential misclassification. Furthermore, subjects were selected from a single medical school, though we have no reason to think that our institution would be different from other universities. In order to improve generalizability, future research could involve other universities.

Other limitations that need to be considered potentially affect the internal validity of our results. Most of these threats or limitations arose outside of the investigators’ control from the nature of the educational setting. An instrumentation-like threat was discovered in our study that we think may have threatened the internal validity of our results, leaning in favor of the control group. There were 2 significant changes in scoring the M-OSCE outcome measure for the experimental group that were outside of the investigators’ control. A different score sheet that included a more precise set of grading instructions could have contributed to a difference in grading the M-OSCE between the 2 years. Also, standardized patients were only used to grade the experimental group, whereas an untrained third-party observer was used to grade the control group. These changes may have led to a harsher grading system for the experimental group. Specifically, we suspect that these changes in scoring and grading systems likely resulted in a differential misclassification of M-OSCE skills scores such that the experimental group received generally lower scores than the control group. For these reasons we suspect that had it not been for the change in grading system, the experimental group would have performed better than the control group. Further, this issue of unforeseen changes in the midst of an investigation is an important area for educational researchers to be aware of when conducting an experimental study.

Another limitation is related to the number of questions used to assess the knowledge outcome, which was limited to 3 for each version of the MCE due to time constraints. Many students from the experimental group answered a single question regarding hip pain incorrectly and consistently picked the same wrong answer (which implied referred pain to the knee). Only the students in the experimental group were exposed to a history threat that may have influenced their response to this question. During this groups’ review lecture for their OSCE testing, another faculty member (who was unaware of and uninvolved with our study) reviewed the musculoskeletal portion of the OSCE and specifically stressed the topic of hip pain (including presenting signs and symptoms) due to poor performance in previous years. This suggests that there may be measurement error for the knowledge assessment. In fact, when this question was eliminated there was still no difference in knowledge score between the 2 classes, suggesting that the curriculums were equally efficacious with regard to knowledge.

The qualitative data obtained suggest that we addressed some of the findings of the needs assessment. After a change in the curriculum, the experimental curriculum group did as well as the traditional curriculum group with the added benefit of overall satisfaction and the addition of a new resource to teaching. Unfortunately, the MCE and M-OSCE scores did not suggest that knowledge or skills improved with the interventional curriculum. These results may be related to the limitations described above or that we need to fine-tune our intervention and testing instruments. Future physicians and patients can benefit from improvements in medical school curricula. Useful feedback was obtained regarding student and faculty satisfaction that can be applied to improve both this curriculum as well as other web-based curriculum aimed at teaching musculoskeletal physical diagnosis skills to medical students. Specifically, this feedback includes shortening and streamlining the content, further facilitator preparation, more time for peer practice, and improving the testing instruments. Students appreciated having a persistent reference that they can view according to their time schedule, and a website affords that luxury.

## References

[1] U.S. Department of Health and Human Services. (2000). Healthy people 2010: understanding and improving health.

[2] Doherty M, Lanyon P (2000). Rheumatology: what should all doctors know?. Ann Rheum Dis..

[3] Walker DJ, Kay LJ (2002). Musculoskeletal examination for medical students: the need to agree what we teach. Rheumatology (Oxford).

[4] (2000). The future status of pediatric rheumatology in the United States: strategic planning for the year 2000. American College of Rheumatology Blue Ribbon Committee for Academic Pediatric Rheumatology. Arthritis Rheum..

[5] Lillicrap MS, Byrne E, Speed CA (2003). Musculoskeletal assessment of general medical in-patients–joints still crying out for attention. Rheumatology (Oxford)..

[6] Branch VK, Graves G, Hanczyc M, Lipsky PE (1999). The utility of trained arthritis patient educators in the evaluation and improvement of musculoskeletal examination skills of physicians in training. Arthritis Care Res..

[7] Branch VK, Lipsky PE (1999). The use of trained arthritis patients in the instruction of the musculoskeletal examination. J Rheumatol Suppl..

[8] Roberts C (2002). Improving the quality of care of musculoskeletal conditions in primary care. Rheumatology (Oxford)..

[9] Dacre JE, Fox RA (2000). How should we be teaching our undergraduates?. Ann Rheum Dis..

[10] Haq I, Dacre J (2003). Computer-assisted learning in undergraduate and postgraduate rheumatology education. Rheumatology (Oxford)..

[11] Day CS, Yeh AC, Franko O, Ramirez M, Krupat E (2007). Musculoskeletal medicine: an assessment of the attitudes and knowledge of medical students at Harvard Medical School. Acad Med..

[12] Hassell AB, West Midlands Rheumatology Services and Training Committee (2002). Assessment of specialist registrars in rheumatology: experience of an objective structured clinical examination (OSCE). Rheumatology (Oxford)..

[13] Schmale GA (2005). More evidence of educational inadequacies in musculoskeletal medicine. Clin Orthop Relat Res..

[14] Freedman KB, Bernstein J (1998 Oct). The adequacy of medical school education in musculoskeletal medicine. J Bone Joint Surg Am..

[15] Association of American Medical Colleges. (2005). Contemporary issues in medicine: musculoskeletal medicine education.

[16] Beattie KA, Bobba R, Bayoumi I, Chan D, Schabort I, Boulos P (2008). Validation of the GALS musculoskeletal screening exam for use in primary care: a pilot study. BMC Musculoskelet Disord..

[17] Smith MD, Walker JG, Schultz D, Ash J, Roberts-Thomson P, Shanahan EM (2002). Teaching clinical skills in musculoskeletal medicine: the use of structured clinical instruction modules. J Rheumatol..

[18] Quellmalz ES, Pellegrino JW (2009). Technology and testing. Science.

[19] Berman NB, Fall LH, Maloney CG, Levine DA (2008). Computer-assisted instruction in clinical education: a roadmap to increasing CAI implementation. Adv Health Sci Educ Theory Pract..

